# Decoding the functional plasticity of milk-derived exosomes: implications for nutrition, immunity, and therapy

**DOI:** 10.3389/fimmu.2025.1645355

**Published:** 2025-10-14

**Authors:** Shahid Hussain, Sundas Ijaz, Abdul Wajid, Abdul Qadeer, Muath Suliman, Fuad M. Alzahrani, Khalid J. Alzahrani, Khalaf F. Alsharif, Chieh-Wei Chang, Chien-Chin Chen

**Affiliations:** ^1^ Department of Biotechnology, Kohsar University, Murree, Pakistan; ^2^ Faculty of Pharmacy, Gomal University, Dera Ismail Khan, Khyber Pukhtunkhwa, Pakistan; ^3^ Department of Cell Biology, School of Life Sciences, Central South University, Changsha, China; ^4^ Department of Clinical Laboratory Sciences, College of Applied Medical Sciences, King Khalid University, Abha, Saudi Arabia; ^5^ Department of Clinical Laboratories Sciences, College of Applied Medical Sciences, Taif University, Taif, Saudi Arabia; ^6^ Division of General Surgery, Department of Surgery, Ditmanson Medical Foundation Chia-Yi Christian Hospital, Chiayi, Taiwan; ^7^ Department of Pathology, Ditmanson Medical Foundation Chia-Yi Christian Hospital, Chiayi, Taiwan; ^8^ Department of Cosmetic Science, Chia Nan University of Pharmacy and Science, Tainan, Taiwan; ^9^ Doctoral Program in Translational Medicine, National Chung Hsing University, Taichung, Taiwan; ^10^ Department of Biotechnology and Bioindustry Sciences, College of Bioscience and Biotechnology, National Cheng Kung University, Tainan, Taiwan

**Keywords:** milk, exosomes, immunity, miRNAs, MDEs

## Abstract

Through the targeted release of immunologically active cargo, milk-derived exosomes (MDEs) are becoming increasingly important channels for maternal-neonatal communication. This study summarizes available data, showing that the bioactivity of MDEs varies and is significantly influenced by factors such as species origin and lactation stage (colostrum versus mature milk). It is argued that this functional variability presents both opportunities and challenges for developing therapeutics and is crucial for understanding their role in shaping the newborn’s immune system. The composition of colostrum-derived MDEs differs significantly from that in mature milk, although both are rich in immunomodulatory microRNAs (such as miR-181a and miR-155) and proteins that promote immune tolerance and gut barrier integrity. Furthermore, the importance of careful source selection is highlighted by interspecies differences in MDE cargo, such as the varying anti-inflammatory properties of camel versus bovine exosomes. To address major challenges like standardization and scalable production, the potential of MDEs as natural nano-carriers for immunomodulatory therapy was critically evaluated. This review offers a framework for future research in nutritional immunology, moving beyond a simple component list to critically assess source-dependent functionality.

## 1 Introduction

The evolutionarily conserved lipid-bound nanocarriers known as extracellular vesicles (EVs), like exosomes, allow for intercellular communication by delivering functional cargo to recipient cells, such as proteins, DNA, and various RNA species (1, 2). This affects a broad range of physiological and pathological processes, from immune modulation to cancer metastasis (3, 4). These vesicles, which contain a wide variety of bioactive compounds, are especially plentiful and persistent in milk-derived exosomes (MDEs), which have been recovered from bovine and human species (5). The dynamic nature of MDEs’ payload, which is specifically adapted to the neonate’s developmental requirements, is a crucial component. For example, immunomodulatory microRNAs (like miR-181a and miR-155) and proteins (such as lactadherin and immunoglobulins) that are essential for training the developing immune system and maintaining the integrity of the gut barrier are greatly abundant in colostrum-derived MDEs (6). Higher quantities of proteins linked to apoptosis and cell motility, on the other hand, indicate that the profile of mature milk MDEs changes towards a cargo supporting tissue maturation, metabolic regulation, and cellular homeostasis (7). The complex biological role of MDEs is highlighted by this functional plasticity, which is further varied by species-specific adaptations (8). Their isolation, typically achieved through methods such as ultracentrifugation (9), exposes 30–300 nm nanoparticles that are well-suited for cellular absorption and systemic dispersion, underscoring their immense potential as nutritional immunomodulators (10) and natural therapeutic agents (11). Although it is commonly known that MDEs include a variety of biomolecular cargo, a comprehensive review of the literature reveals a more complex story (12). MDEs’ immunological effects vary greatly depending on their biological setting and are not a general characteristic (13). The lactation stage, where colostrum MDEs are primed for immune education and mature milk MDEs may support tissue growth and homeostasis, and the species of origin, which confers unique functional characteristics on their exosomal cargo, are two factors that stand out as being particularly decisive (14). Therefore, using this functional plasticity as a perspective, this review will critically evaluate the evidence supporting MDEs as immunomodulatory drugs. In addition to discussing the substantial translational challenges posed by their intrinsic heterogeneity, the implications of this diversity for their inherent role in infant health and their potential as therapeutic vehicles are explored. Although the literature now in publication thoroughly lists the various bio-molecular cargoes of MDEs, a purely descriptive approach restricts their potential for therapeutic use (15). This review goes beyond a synopsis to offer a critical synthesis, suggesting that a framework of source-dependent functional heterogeneity governs the bioactivity of MDEs, which is not uniform. We have critically assessed the data, which reveals that two key factors species-specificity and lactation stage (colostrum vs. mature milk) create a range of MDE effects, from tissue healing to strong immunomodulation.

## 2 Role of milk-derived exosomes in neonatal health

### 2.1 Exosome composition in mature milk and colostrum

EVs derived from human milk mainly consist of proteins, lipids, and DNA, which are typically released by cells (16). Research has identified about 639 proteins and peptides within these EVs. Additionally, both term and preterm human MDEs contain 395 different lipids. Notably, up to 50 of these lipids are involved in regulating the activity of intestinal epithelial cells (17). The composition of MDEs varies greatly between colostrum and mature milk, as summarized in **Table 1**, with functional implications discussed in Section 4.

**Table 1:** Comparative composition of exosomes derived from colostrum and mature milk.

Component Type	Mature Milk MDEs	Colostrum MDEs	References
Lipids	Greater diversity of phospholipids and glycerolipids associated with metabolism	Higher levels of sphingomyelin and phosphatidylserine (linked to immune signaling)	(17)
lncRNAs	Mature milk contains more lncRNAs related to apoptosis and differentiation	Stage-dependent variation; colostrum MDEs enriched with immune-modulatory lncRNAs	(17)
miRNAs	More stable expression of growth/metabolic miRNAs	Higher abundance of immune-regulatory miRNAs (e.g., miR-181a, miR-155, miR-223)	(18)
Metabolites	More metabolites involved in energy metabolism and gut maturation	Richer in bioactive metabolites that support neonatal immune development	(19)
Proteins	Higher levels of proteins linked to apoptosis, cell motility, metabolic regulation	Enriched in immune-related proteins (e.g., immunoglobulins, lactoferrin, antimicrobial peptides, inflammatory proteins)	(20)

Nucleic acids have attracted considerable interest among exosome components due to their significant role in regulating metabolic processes (21). Milk exosomes contain a wide variety of nucleic acids, including deoxyribonucleic acid (DNA), mRNA, miRNAs, circular RNAs, and long non-coding RNAs (lncRNAs). In particular, milk is a rich source of miRNAs (22). Lipidomic research of MDEs has identified several common lipids, such as sphingomyelin, phosphatidylcholine, phosphatidylserine, and phosphatidylethanolamine (19). Exosomes may affect the function of the mammary gland. Comparing the proteome of highly purified milk exosomes with that of whole milk can uncover the actual protein content of these exosomes. Similar analyses can be performed on EVs from other body fluids. Studying the proteome of MDEs could provide insights into their potential medical uses, such as biocompatible drug delivery systems or tools for personalized therapy. Some of these applications are summarized in **Table 2**.

**Table 2:** Applications of MDEs for the treatment of several diseases.

MDEs origin	Study Model	Major outcomes	Molecular mechanism/exosomal cargo	References
Camel	Breast cancer cells and Albino rats	Anti-cancer activity and Antioxidant activity	Delivery of pro-apoptotic proteins and miRNAs (increase Bax, caspase-3, decrease Bcl-2); reduces oxidative stress	(23)
Yak	IEC-6(intestinal epithelial cell line)	create tolerance for hypoxia	Regulation of HIF-1α and VEGF pathways; delivery of growth-promoting miRNAs	(24)
Human	Monocyte-derived dendritic cells (MDDCs) and CD4+ T cells derived from intestinal organoids	Anti-HIV-1 inflammatory-reduction capacity	miR-146a and miR-21 in exosomes modulate NF-κB signaling and cytokine secretion	(25)
Porcine	Neonatal unbuckled piglet (IPEC-J2) *in vitro* jejunum Blood T lymphocytes, cultured	Intestinal cell proliferation Immunity and digestive tract development in neonatal piglets	miR-181a, miR-155, and miR-223 regulate T-cell development and intestinal gene expression	(18, 26)
Bovine	Mice, Goblet cell	Attenuates Arthritis Growth of rheumatoid necrotizing enterocolitis and immune response	Exosomal TGF-β, miR-148a, and miR-320 suppress proinflammatory cytokines; enhance goblet cell mucin secretion	(27)
Buffalo	Bioinformatics	avoiding infections and inflammatory diseases	miR-26a, miR-30a target genes linked to inflammation and infection pathways	(28)
Goat	Mice	inflammatory-decreasing qualities	Exosomal miR-21, miR-146 regulate TLR4/NF-κB signaling	(14)
Sheep	Bioinformatics	Immune response & inflammation during infection	Exosomal miRNAs predicted to target host immune-related genes	(29)
Rat	Intestinal epithelial cells	Anti-Necrotizing Enterocolitis	miR-148a and TGF-β in exosomes reduce apoptosis and ER stress	(30)

### 2.2 Biological functions of MDEs in neonatal health

**Figure 1:**
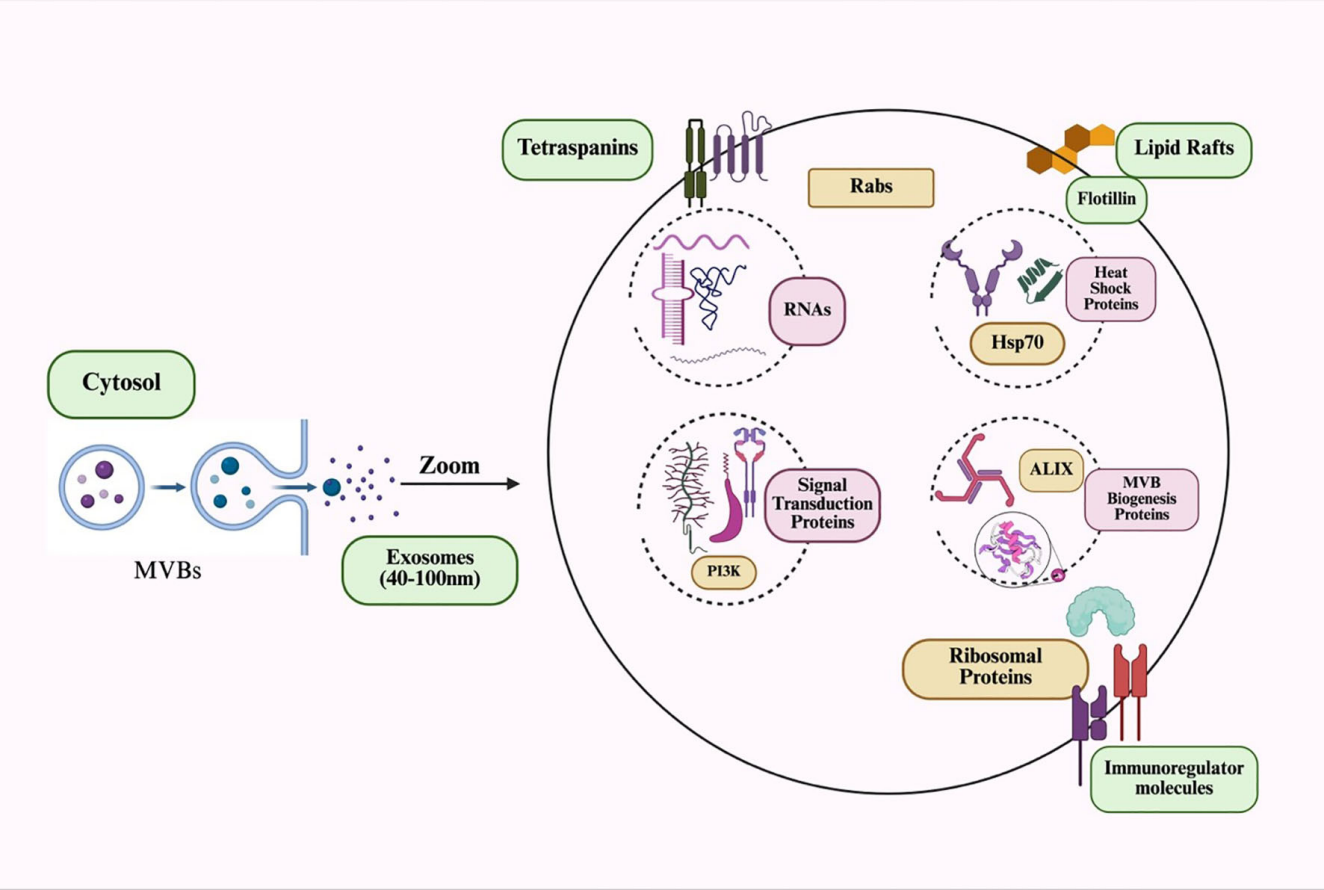
Graphical representation of MDEs and their general cargos component.

**Figure 2:**
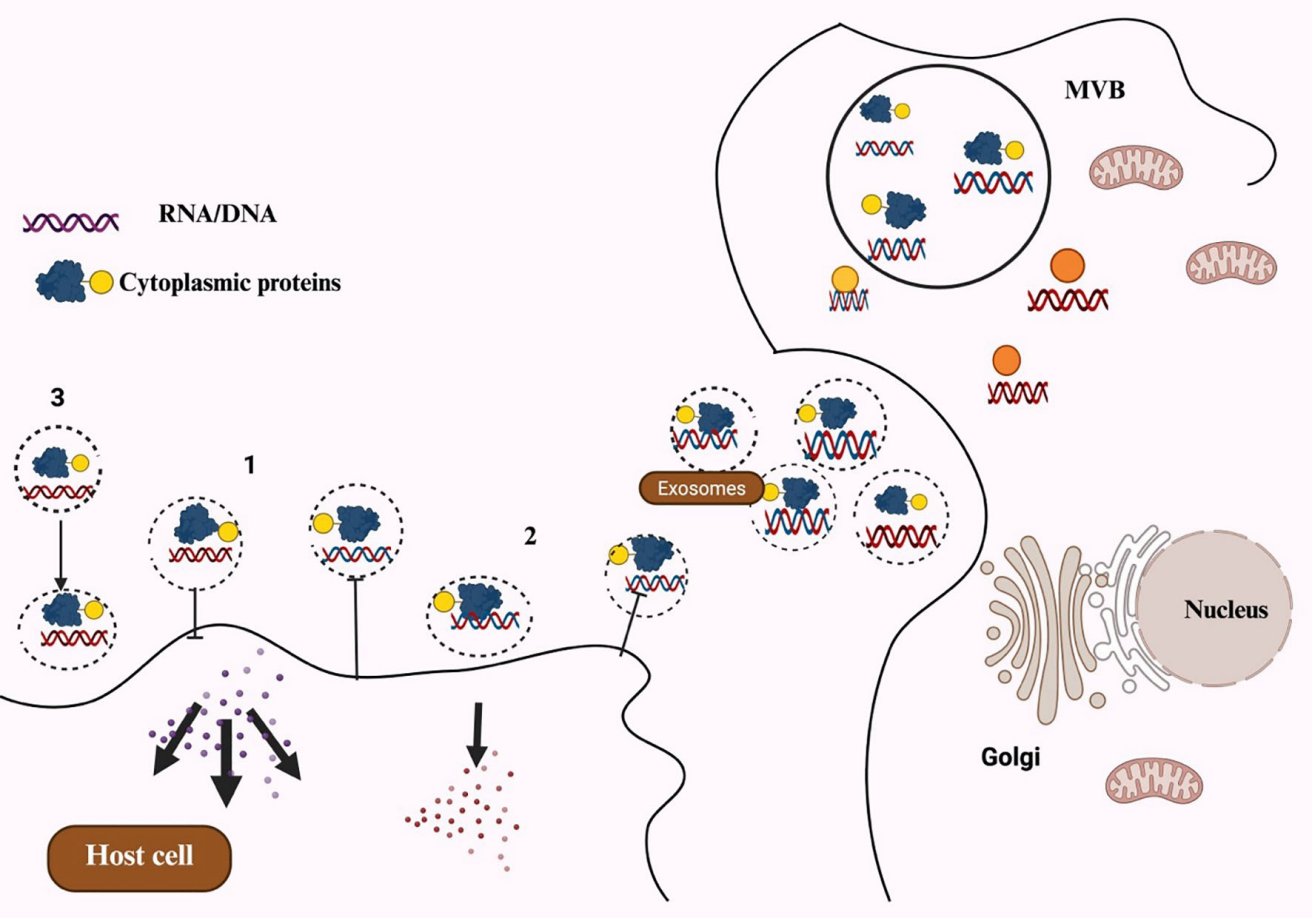
Schematic representation of exosomes-mediated cell-to-cell communication channels: (1) Recipient cells are signaled by exosomes directly through surface-bound ligand. (2) Activated receptors are delivered to recipient cells via exosomes. (3) Exosomes can transfer functional lipids, proteins, and RNAs to recipient cells, which could epigenetically remodel cells.

One of the most important signaling particles that facilitates cellular communication between mother and child is MDEs. These exosomes play a key role in protecting newborns from conditions such as inflammatory bowel disease, diabetes, and obesity. They also boost the child’s immune system through the antibodies they contain. Additionally, human breast milk includes other crucial components, such as immune cells, soluble proteins like IgA, cytokines, and antimicrobial peptides, all of which help defend newborns against early illnesses (31). It also inhibits the proliferation of various cell lines (32). The general exosome cargos are shown in **Figure 1**. The cell-to-cell communication mediated by exosomes is illustrated in **Figure 2**.

### 2.3 Potential applications of milk-derived exosomes in pediatric medicine

Due to their diverse biomolecular cargo, MDEs are rich in several medically essential molecules, as detailed in **Table 3**. These exosomes may help prevent the death of intestinal epithelial cells, offering a promising therapeutic option for children with intestinal damage. Since necrotizing enterocolitis (NEC) is a major cause of morbidity and mortality in newborns, MDEs present a potential treatment to reduce the incidence and severity of NEC in at-risk infants (39). Cow’s milk exosomes have been shown to help prevent NEC in test mice by enhancing goblet cell mucin expression, increasing the number of goblet cells, and improving endoplasmic reticulum (ER) function (40). Human breast milk is known to support blood clotting (41).

**Table 3:** List of common proteins derived from milk exosomes.

Source of milk exosomes	Most represented proteins	Proteins described	Method for analysis of proteins	References
Cow	Xanthine Butyrophilin oxidase Adipophilin Lactadherin	2107	Liquid Chromatography-MS/MS, Trypsinolysis	(33)
Human	Tenascin, Serum albumin -casein, Lactoferrin, and xanthine dehydrogenase Ig polymer receptor	115	Liquid Chromatography-MS/MS, Trypsinolysis	(34)
Cow	Xanthine Butyrophilin dehydrogenase fatty acid synthase Lactadherin	2299	iTRAQ-LC-MS/MS, Trypsinolysis	(35)
Human	Syntenic G-protein subunits CD9, CD63, CD81, Flotilin, Lactadherin, and Annexin proteins linked to Ras Rab	2698	Trypsinolysis, SDS-PAGE, and LC-MS/MS	(36)
Horse	Lacto globulin Lactadherin Actin Butyrophilin Lactoferrin	08	SDS-PAGE, 2D electrophoresis, MALDI-TOF-MS/MS, and Trypsinolysis	(37)
Swine	Albumin, Lacto transferrin, Ceruloplasmin, Thrombospondin, Complement C4, -Glucosidase	571	Trypsinolysis, SDS-PAGE, and LC-MS/MS	(38)

### 2.4 Applications of MDEs in wound healing

Bovine milk exosomes positively affect the three main types of skin cells—keratinocytes, melanocytes, and fibroblasts—by reducing UV-related aging and damage. They help prevent the buildup of intracellular reactive oxygen species and UV-induced oxidative stress in epidermal keratinocytes. As a result, bovine milk exosomes have significant potential as a natural therapeutic agent for reversing UV-related skin aging and damage (42).

### 2.5 Challenges and limitations

Considering the promising applications of MDEs in neonatal care, several issues need to be addressed. Standardization is difficult due to the wide variation in MDE composition across species, individuals, and lactation stages. Exosomal integrity and functional biomolecules might be compromised by industrial processes like pasteurization, which could diminish their therapeutic value in baby formula. Additionally, there is a lack of robust clinical data in humans, despite animal studies demonstrating benefits against diseases such as intestinal inflammation (43).

## 3 Comparative analysis and functional heterogeneity of milk-derived exosomes

A review of the literature shows that the functional power of milk-derived exosomes (MDEs) varies depending on their biological context, especially the stage of lactation and the species they originate from (7). Colostrum-derived MDEs are rich in immunomodulatory elements (such as miR-181a, lactoferrin) that act as immune triggers for the newborn, while MDEs from mature milk primarily support tissue development and help maintain balance (44). Additionally, comparisons across different species reveal a functional toolkit: bovine MDEs excel at protecting the gut barrier (45), camel MDEs have potent anticancer effects (46), and goat MDEs are highly anti-inflammatory (47). This diversity emphasizes that MDEs are a varied group of biologics; therefore, a one-size-fits-all approach is ineffective. Future studies and therapies should carefully select MDE sources based on their specific functions to ensure effectiveness and consistency.

## 4 MDEs in cancer therapy

### 4.1 Overview of exosome-mediated intercellular communication in cancer

**Figure 3:**
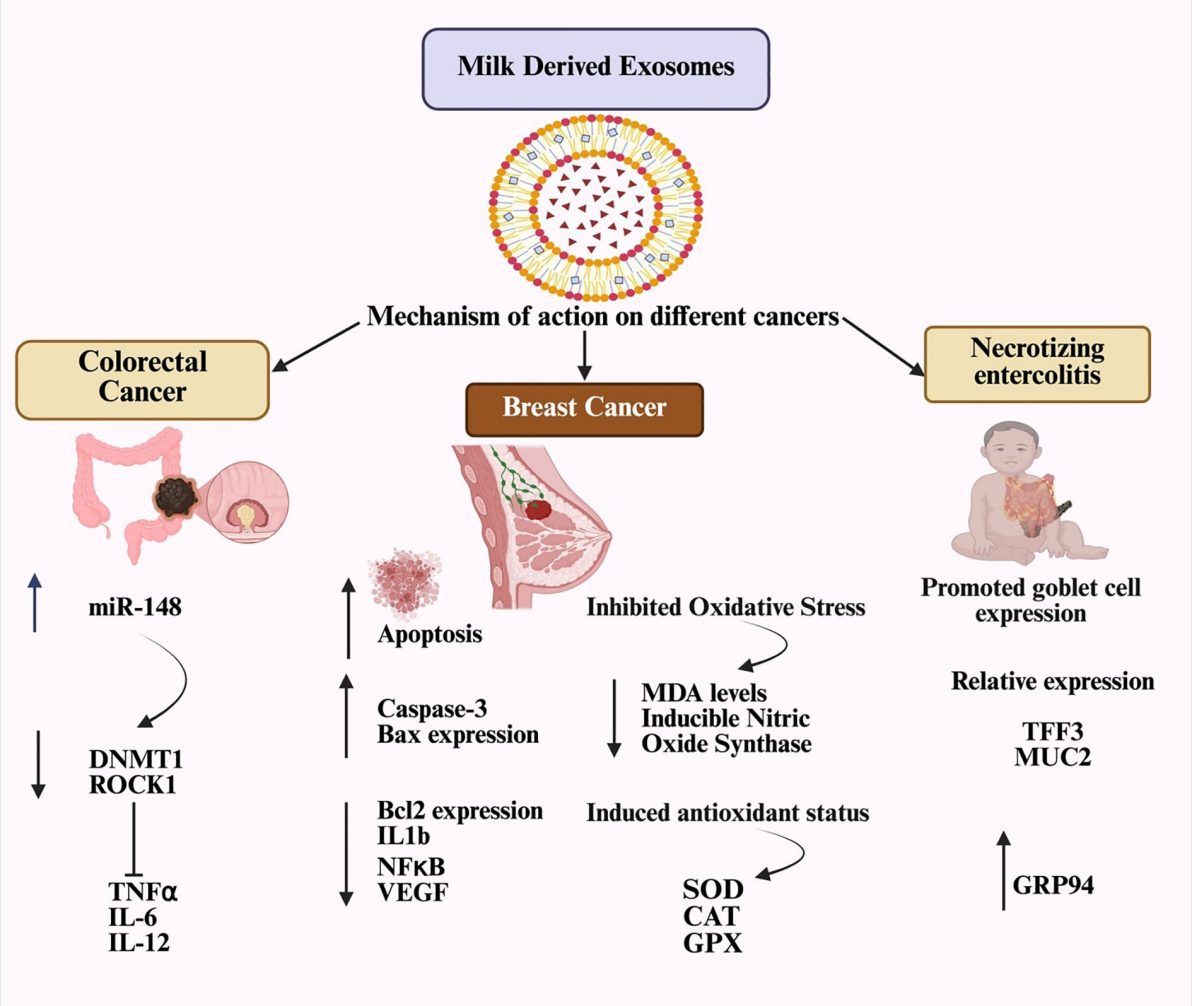
The Mechanism of MDEs has multiple effects on disease.

Exosomes are involved in thrombosis, cancer cell growth, extracellular matrix remodeling, and angiogenic stimulation. Their high stability supports tumor environments, aiding the development of metastatic niches (44). Exosome-mediated communication allows the transfer of messages to various target sites. Tumor-released exosomes can passively travel through the bloodstream and bodily fluids, where they bind to the extracellular matrix. Despite their widespread distribution, exosomes have a very short half-life in circulation, with nearly 90% being eliminated within five minutes of infusion (45). The *in vivo* biodistribution of exosomes is affected by factors such as the target cells, delivery method, and their origin. Recipient cells internalize exosomes through receptor-mediated endocytosis, membrane fusion, or other mechanisms. The ways in which MDEs influence specific diseases are shown in **Figure 3**.

### 4.2 Strategies for delivering exosomes to cancer cells

Exosomes can enhance the invasive and metastatic abilities of recipient cells, promote epithelial-mesenchymal transition (EMT), and contribute to matrix remodeling and the formation of metastases. They play a vital role in angiogenesis, highlighting their significance in the progression of gastrointestinal cancers. Tumor-derived exosomes utilize various mechanisms to stimulate angiogenesis and support tumor growth (46, 47). Anti-cancer therapeutic exosomes can target cancer cells or tissues either passively or actively. Natural tropism allows for the passive targeting of exosomes, while active targeting is achieved through surface modifications of exosomal membranes using different technical methods. Passive targeting is well-established; nanoparticles smaller than 100 nm can be delivered to the tumor parenchyma via the “enhanced permeability and retention” (EPR) effect (48). Exosomes may have inherent tumor-targeting abilities depending on their cell of origin. In active targeting, exosomes can be directly engineered on their surface with various external methods to specifically target and deliver anti-cancer therapies to tumor cells. Additionally, exosomes can be indirectly engineered by genetically modifying the cells from which they originate (49–51).

### 4.3 Preclinical studies on the efficacy of milk-derived exosomes in cancer therapy

Exosomes from tumor cells have been used to treat pleural effusion and malignant ascites (52). Breast and lung cancers showed better responses to chemotherapeutic drugs delivered through exosomes from buffalo milk. The potential for future oral chemotherapy could be increased by the ability of exosomes from bovine milk to cross the gastrointestinal barrier (53). Exosomes carrying chemotherapeutic drugs may accumulate excessively in various tissues through passive targeting, which could pose risks to the liver, kidneys, or heart, as summarized in **Table 4**. However, milk-derived exosomes modified with folic acid enhance both the effectiveness and safety of cancer drugs, especially in cancers with high folic acid receptor expression (54).

**Table 4:** MDEs from different animals worked as a carrier for effective drugs.

Exosome source	Model system	Therapeutic cargo	Key findings
Camel MDEs	Breast cancer in rats	Native (no drug)	54% tumor growth reduction, increase apoptosis, decrease oxidative stress (23)
Bovine MDEs	Breast cancer xenograft (mouse)	Doxorubicin	2.5× higher tumor drug accumulation vs free drug (54)
Bovine MDEs	Lung cancer xenograft (mouse)	Paclitaxel	Approx. 3× higher tumor inhibition, reduced systemic toxicity (53)
Bovine MDEs	NSCLC xenograft (mouse)	Celastrol	Significant tumor suppression, no chronic toxicity (55)
Buffalo MDEs	Breast and lung cancer (*in vitro* & *in vivo*)	Chemotherapeutics	Improved sensitivity to drugs (22)

### 4.4 Challenges and strategies in MDE-based cancer therapy

Attaching anticancer medications to the surfaces of naturally occurring, physiologically active structures such as proteins significantly enhances the biological availability and effectiveness of the therapy (56). The development of exosomes as medicinal agents faces several challenges. Collecting exosomes from clinical models is impractical for large-scale pharmaceutical production, and when administered systemically, the protein components of exosomes are likely to provoke immune responses (57, 58). MDEs were introduced into mouse models, and they did not cause systemic toxicity or anaphylactic reactions (59). Non-loaded camel milk exosomes notably inhibited breast cancer growth, as shown by increased apoptotic markers, decreased oxidative stress, and downregulation of several genes related to inflammatory mechanisms and immune response activation (23). In a xenograft model of non-small cell lung cancer, celastrol-loaded milk exosomes showed significantly greater antitumor activity compared to free celastrol. Delivering celastrol via milk exosomes did not result in significant chronic toxicity (55). One challenge with using milk exosomes for targeted drug delivery is their lack of specificity for recipient cells. Whole milk exosomes are absorbed from the gut and can be modified with ligands to improve their retention in target tissues (60). Specific ligands can be incorporated into milk exosome-based vectors to target tumor-specific receptors. For example, the lipid membrane of milk exosomes can be modified with hyaluronan molecules to enable targeted delivery of the cytostatic drug doxorubicin to cells expressing the CD44 receptor. Many cancer cells exhibit high levels of CD44 and its ligand, hyaluronan (61). *In vitro* studies showed that bovine milk exosomes activate CD69 on normal killer (NK) cells. This activation may unintentionally boost inflammatory processes, as NK cells and T lymphocytes produce increased levels of interferon (IFN) when co-activated with milk exosomes and interleukins 2 and 12 (62). The use of milk exosomes for oral delivery of therapeutic agents holds great potential, as it can significantly improve the efficacy of anticancer drugs while reducing therapy-related toxicity (54).

## 5 Milk-derived exosomes in tissue regeneration

**Figure 4:**
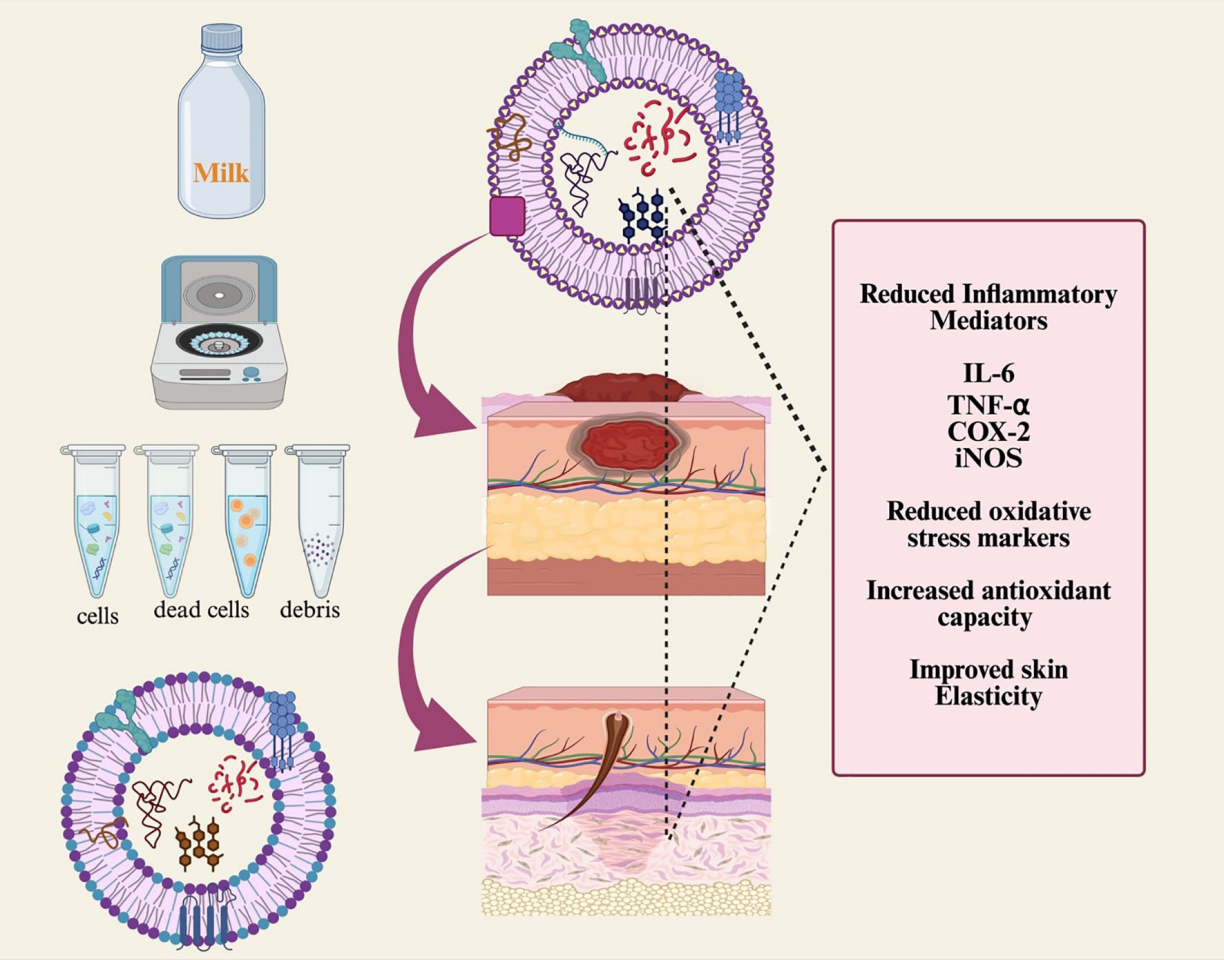
Schematic diagram showing milk-derived exosome isolation and its biological role in wound healing. Cancer susceptibility of gene 101 (TSG101), cyclo-oxygenase (COX-2), and milk EXO isolation and its impact on wound healing.

Exosomes are among the most effective methods for wound healing because of their biocompatibility, origin from healthy cells, ability to modulate inflammatory responses, and capacity to promote cell growth and migration (63). TGF-β3 and TGF-β1 are known to play vital roles in wound healing. MDEs have been reported to inhibit cell migration in the intestinal epithelial cell line IEC-18. MDEs are especially promising for treating various types of scars and keloids, including those from skin injuries, acne, abrasions, and surgical incisions (64). Bovine milk-derived exosomes have shown beneficial effects in reducing ultraviolet-induced skin aging and degeneration across three skin cell types: keratinocytes, melanocytes, and fibroblasts. Milk exosomes can inhibit the production of intracellular reactive oxygen species and UV-induced damage in epidermal keratinocytes. They also decrease melanin production in UV-stimulated melanocytes, potentially addressing hyperpigmentation disorders. Furthermore, milk exosomes have been shown to lower matrix metalloproteinase expression in human endothelial cells, indicating their significant potential as a natural therapy for reversing ultraviolet-induced skin aging and damage (65). The effects of exosomes on wound healing are shown in **Figure 4**.

## 6 Milk-derived exosomes in immunomodulation

Many components in breast milk, including immunoglobulins, oligosaccharides, glycoproteins, maternal cells, and probiotic bacteria, have immunoregulatory properties that may influence their overall effects. Exosomes in human breast milk interact with peripheral blood mononuclear cells (PBMCs) to boost IL-5 production while decreasing the synthesis of IL-2, IFN-γ, and TNF (66). Milk exosomes carry a significant amount of miRNA with potential immunomodulatory effects (17). MiR-148a helps regulate the functions of B and T lymphocytes and may also contribute to the prevention of autoimmune and inflammatory diseases. Recent studies show that mature bovine milk exosomes and colostrum contain the highest concentrations of miRNAs linked to the immune system, including miR-181a, miR-26a, and miR-19 (67). Therefore, miRNA in milk exosomes from different species supports the development of the fetus’s immune system. MDEs can transfer genetic material from mother to child, influencing the baby’s immune response, which is vital for treating various disorders. This is most notably seen with camel-derived milk exosomes (68). Camel milk proteins offer several benefits, including immunomodulatory and antioxidant properties. They are especially effective in regulating inflammatory responses and boosting immune reactions in species treated with cyclophosphamide, as they help reduce oxidative stress and enhance antioxidant defenses (22). Besides immune cells, human breast milk contains soluble proteins, including cytokines, IgA, antimicrobial peptides, and other substances (69). Both mature human breast milk and animal milk contain exosomes expressing tetraspanin proteins CD63 and CD81, along with the MHC class II protein CD86 (54). MDEs can decrease cytokine production by PBMCs stimulated by anti-CD3 and promote the expansion of Foxp3+ CD4+/CD25+ T regulatory cells. As a result, these exosomes can influence a child’s immune system (66). Porcine milk exosomes from pig milk contain various bundled miRNAs and play a vital role in piglet growth. These components significantly impact the regulation of the immune system and the development of the digestive tract in newborn piglets (18). Although breastfeeding can transmit HIV-1 from mother to child, the risk of transmission is less than 30%.

### 6.1 Clinical trials on the efficacy of MDEs in immunomodulation

We examined the effects of Col-exo, a component of bovine milk, on a murine model of ulcerative colitis induced by dextran sodium sulfate (DSS). Col-exo effectively neutralized reactive oxygen species and modulated immune cytokine production, promoting the growth of colonic epithelial cells and macrophages in an anti-inflammatory environment. Additionally, Col-exo can pass through the digestive system intact, delivering bioactive substances to the stomach, small intestine, and colon. Our results suggest that oral administration of Col-exo can alleviate colitis symptoms such as weight loss, intestinal bleeding, and prolonged diarrhea by regulating duodenal inflammatory immune responses. Overall, the robust structural and functional stability of bovine colostrum-derived exosomes highlights their potential as a natural treatment for wound healing (70). Human breast milk (HBM) contains a diverse array of components, including a microbiome, EVs, and miRNAs, in addition to its nutritional content and non-nutritional proteins, such as hormones, growth factors, and immunoregulatory proteins. Milk-derived exosomes have demonstrated a wide range of physiological and therapeutic effects on cancer, inflammation, and cell proliferation, primarily due to the proteins and microRNAs they contain. Exosomal miRNAs play a crucial role in immune regulation and tumor development, as they are resistant to enzymatic digestion and acidic conditions. Moreover, research explores the use of milk-derived exosomes as drug delivery systems for siRNA and small molecules targeting tumor sites (71). Exosomes derived from milk, citrus pectin, and dietary omega-3 polyunsaturated fatty acids can reduce inflammation at the intestinal barrier. Their molecular actions primarily include enhancing the expression of tight junction proteins, promoting epithelial cell proliferation, enriching the mucus layer, modulating immune responses, and preventing inflammatory cell infiltration (72). The findings showed that the bidirectional immunomodulatory effects of EVs from various dairy products were similar to those of EVs from raw milk. These effects included promoting normal macrophage proliferation, increasing NO and cytokine levels, and inhibiting the LPS-induced TLR4/NF-κB pathway, as well as reducing inflammatory cytokine production. Notably, dairy-derived EVs can alter the expression of miR-155, miR-223, and miR-181a, which are crucial for the body’s response to infection (73).

### 6.2 Challenges and future direction in milk-derived exosomes-based immunomodulation

Due to the molecular complexity of MDEs, many obstacles hinder their clinical translation as immunomodulatory drugs. The primary challenge is the high heterogeneity in exosomal formulations, where significant variations in cargo and bioactivity result from species differences, individual donor variability, and most notably, the stage of lactation (such as colostrum versus mature milk) (74). Calculating dosage and validating efficacy are further complicated by the difficulty in replicating therapeutic effects from a single MDE source, due to a lack of standardized composition (75). A major barrier to their use in baby formula or biotherapeutics is that industrial processing steps, such as pasteurization, can destroy sensitive immunomodulatory cargos, including specific miRNAs and proteins, which are crucial for their therapeutic effects and thereby compromise exosomal integrity (76). Future research must focus on several key areas to overcome these challenges and unlock the unique epigenetic and immunoregulatory potential of MDEs. First, the field needs to establish strict, potency-based quality control measures that go beyond simple particle counting. This involves developing standardized assays to assess the levels and integrity of important functional components, such as immunomodulatory proteins or miRNAs (e.g., miR-148a) (77). Second, standards for Good Manufacturing Practice (GMP) tailored specifically for MDEs must be developed promptly. These standards should ensure batch-to-batch consistency and reliable therapeutic outcomes by considering critical factors, including source species, lactation stage, and processing history (78). Lastly, research should shift from descriptive to mechanistic studies that establish direct links between specific MDE cargos and precise immunological effects. By adopting this focused and standardized approach, the field can unlock the substantial potential of MDEs as next-generation, natural immunomodulators.

### 6.3 Clinical translation and future challenges

Even with strong preclinical potential, numerous obstacles remain to be overcome before MDEs can be utilized in clinical settings. Critical limitations include a significant lack of data from human trials, unclear regulatory procedures for standardization, and insufficient safety profiles regarding systemic and long-term immunogenicity (79). To evaluate clinical viability, scalable production that complies with Good Manufacturing Practices (GMP) and a comprehensive cost-benefit analysis in comparison to synthetic Nano carriers are also necessary. To fully utilize the therapeutic promise of MDEs in human health, these translational issues must be addressed (79).

## 7 Conclusion

Milk-derived exosomes (MDEs) are complex signaling vehicles that play a crucial role in both newborn immunology and maternal-offspring communication. They are much more than just straightforward biomolecule carriers (80). A comprehensive evaluation of the literature, however, shows that their biological and therapeutic roles are significantly shaped by their origin rather than being general. Important factors influencing their activity include the stage of lactation (colostrum MDEs serve as immunological primers, while mature milk MDEs support tissue homeostasis) (81) and the species of origin (each providing unique functional profiles, ranging from the gut-protective effects of bovine MDEs to the anti-inflammatory properties of camel and goat MDEs) (82). This functional variability presents both opportunities and challenges. Although it makes standardization more difficult, it offers a wide range of tools for targeted therapy, whether they are used to improve drug delivery in oncology, promote wound healing, or mitigate necrotizing enter colitis (74). Further research must extend beyond descriptive cataloguing to fully realize this promise. The field urgently needs to conduct direct, head-to-head comparative research and develop standardized, potency-based characterization techniques that account for source variability. The potential of MDEs as natural, efficient, and targeted medicines in nutritional immunology and precision medicine may be realized by accepting this nuanced view of them as a physiologically diverse class of therapies.
